# Analysis of the molecular and biochemical mechanisms involved in the symbiotic relationship between *Arbuscular mycorrhiza* fungi and *Manihot esculenta* Crantz

**DOI:** 10.3389/fpls.2023.1130924

**Published:** 2023-03-07

**Authors:** Yu Gao, Siyuan Huang, Yujie Wang, Hongxin Lin, Zhiyong Pan, Shubao Zhang, Jie Zhang, Wenquan Wang, Shanhan Cheng, Yinhua Chen

**Affiliations:** ^1^ Sanya Nanfan Research Institute of Hainan University, School of Life Science, Hainan University, Haikou, Hainan, China; ^2^ College of Tropical Crops, Hainan University, Haikou, Hainan, China; ^3^ Soil and Fertilizer & Resources and Environment Institute, Jiangxi Academy of Agricultural Sciences, Nanchang, Jiangxi, China; ^4^ College of Horticulture and Forestry of Huazhong Agricultural University, Wuhan, China

**Keywords:** cassava, symbiosis, arbuscular mycorrhiza, transcriptome, metabolome, candidate gene, fungi

## Abstract

**Introduction:**

Plants and arbuscular mycorrhizal fungi (AMF) mutualistic interactions are essential for sustainable agriculture production. Although it is shown that AMF inoculation improves cassava physiological performances and yield traits, the molecular mechanisms involved in AM symbiosis remain largely unknown. Herein, we integrated metabolomics and transcriptomics analyses of symbiotic (Ri) and asymbiotic (CK) cassava roots and explored AM-induced biochemical and transcriptional changes.

**Results:**

Three weeks (3w) after AMF inoculations, proliferating fungal hyphae were observable, and plant height and root length were significantly increased. In total, we identified 1,016 metabolites, of which 25 were differentially accumulated (DAMs) at 3w. The most highly induced metabolites were 5-aminolevulinic acid, L-glutamic acid, and lysoPC 18:2. Transcriptome analysis identified 693 and 6,481 differentially expressed genes (DEGs) in the comparison between CK (3w) against Ri at 3w and 6w, respectively. Functional enrichment analyses of DAMs and DEGs unveiled transport, amino acids and sugar metabolisms, biosynthesis of secondary metabolites, plant hormone signal transduction, phenylpropanoid biosynthesis, and plant-pathogen interactions as the most differentially regulated pathways. Potential candidate genes, including nitrogen and phosphate transporters, transcription factors, phytohormone, sugar metabolism-related, and SYM (symbiosis) signaling pathway-related, were identified for future functional studies.

**Discussion:**

Our results provide molecular insights into AM symbiosis and valuable resources for improving cassava production.

## 1 Introduction

Plants’ adaptation to diverse ecological systems worldwide depends on many factors, among which their ability to develop mutualistic interactions with symbionts plays important roles (Parniske, 2008; Chen et al., 2022). Arbuscular mycorrhiza (AM) is the most widespread symbiotic association between AM fungi (AMF) and plant roots (Bapaume and Reinhardt, 2012; Ho-Plágaro and García-Garrido, 2022b). The majority of landplant species interact with AMF from the Glomeromycotina phylum (Pimprikar and Gutjahr, 2018; Ho-Plágaro and García-Garrido, 2022b). This reciprocal relationship is highly beneficial and reposed on a bidirectional nutrient exchange between fungi and host plants (Ho-Plágaro and García-Garrido, 2022a). Host plants provide AMF with carbon (photosynthates and fatty acids), while in return, fungi supply plants with essential mineral nutrients, principally phosphorus (P) and nitrogen (N) (Parniske, 2008; Püschel et al., 2020; Zai et al., 2021; Ma et al., 2022; Ho-Plágaro and García-Garrido, 2022a). Moreover, AM symbiosis improves plant growth, production, and tolerance to various biotic and abiotic stresses (Sabra et al., 2018; Gao et al., 2020; Zhang et al., 2020; Jajoo, 2021; Lin et al., 2021; Jumrani et al., 2022). Accordingly, AMF have become symbionts of several research interests and are recommended for wide use as bio-fertilizers to enhance crops’ quality and productivity under the ever-changing climate and to ensure sustainable agriculture (Igiehon and Babalola, 2017; Begum et al., 2019).

AM symbiosis development can be divided into four distinct stages (Gutjahr, 2014; Diédhiou and Diouf, 2018). The first stage, pre−contact signaling, consists of cross-talk between the two symbionts *via* diffusible signal molecules (fungi mainly exudate lipo-chitooligosaccharides, while plants strigolactones, STs). The second step, contact between plant roots and fungal hyphae, consists of physical contact between the two partners, followed by the beginning of hyphopodium formation on root surfaces. The third step, the intra−radical proliferation of the fungal hyphae, consists of the penetration and growth of fungal hyphae in the apoplast of the cortex, altering their typical appearance. The last step, arbuscules formation, consists of fungal hyphaes’ penetration and proliferation in the inner cortex, causing colonization surface (Diédhiou and Diouf, 2018). Arbuscules are the main sites of nutrient exchange between the two symbionts, and their development induces *de novo* synthesis of the peri arbuscular membrane to surround the cytoplasmic membrane (Abdallah et al., 2014; Diédhiou and Diouf, 2018). The AMF recognition by the host plants is mediated by a common symbiosis (SYM) signaling pathway, which is partially shared with Rhizobium-legume symbiosis (Bonfante and Genre, 2010; Genre and Russo, 2016). The SYM signaling pathway and symbionts interactions are mainly regulated by an interplay between TFs and phytohormones (auxin, gibberellin, ABA, STs, and ethylene) (Gutjahr, 2014; Diédhiou and Diouf, 2018; Jiang et al., 2018; Müller and Harrison, 2019; Faizan et al., 2020; Liu et al., 2020; Tominaga et al., 2020; Mitra et al., 2021). Among them, GRAS TFs, specifically the genes NSP1 and RAM1, play essential roles (Floss et al., 2013; Nagae et al., 2014; Hohnjec et al., 2015; Rich et al., 2017; Hartmann et al., 2019; Ho-Plágaro et al., 2019; Ho-Plágaro and García-Garrido, 2022b). Arbuscules’ formation causes changes in the expression patterns of many genes in the AM roots, leading to variation in primary and secondary metabolites and production improvement (Ren et al., 2019; Sakamoto et al., 2019; Shtark et al., 2021; Kaur et al., 2022; Mishra et al., 2022; Zhao et al., 2022). For instance, the expression of plant transporter family genes is significantly induced during AM symbiosis (Porcel et al., 2016; Kameoka et al., 2019; Banasiak et al., 2021; Rui et al., 2022). It is shown that AM-induced molecular mechanisms differ according to the species, genotype, AMF type, and growing conditions (Tsiokanos et al., 2022). Therefore, dissecting the AM-induced mechanisms and regulation in diverse plant species will enable the establishment of an efficient agro-biotechnological approach for using AM in sustainable agriculture and improve the economic and quality values of crops.

Cassava (*Manihot esculenta* Crantz) is a perennial shrub that belongs to the Euphorbiaceae family (Blagbrough et al., 2010). Also called yucca or manioc, cassava originated in South America, from where it was subsequently introduced to tropical and subtropical regions of Asia and Africa (Blagbrough et al., 2010; Mombo et al., 2016). Its tuberous roots are valuable food sources in developing countries and are extensively used to produce starch, bioethanol, and other bio-based products, such as medicine, feed, biopolymers, and cosmetics (Li et al., 2017). Among carbohydrate food sources, cassava ranks fourth in the tropics after rice, maize, and sugar cane (Blagbrough et al., 2010). In Asia, cassava drives the rural economy of several countries as it is cultivated by over 8 million farmers (Malik et al., 2020). Accordingly, one of the main breeding objectives in cassava is to improve storage root and starch yield to ensure the availability of food supply in the current situation of the growing population (Sonnewald et al., 2020). Previous studies have demonstrated that AMF inoculation improved cassava root fresh weight, mineral nutrition, total biomass, and productivity (Ceballos et al., 2013; Aliyu et al., 2018). However, the molecular mechanisms involved in AM symbiosis in cassava are unknown. With the availability of genome information on cassava (Wang et al., 2014; Hu et al., 2021), deciphering AM-induced molecular changes will offer important resources to optimize the crop production and tolerance abilities to meet food and market demand. Furthermore, it is demonstrated that cassava and fungal genetic variation and genotype × genotype specifications regulate exchanges between the two partners (Mateus et al., 2019; Savary et al., 2020). Hence, identifying key biochemical markers and candidate genes may facilitate the efficient use of AMF in improving cassava production. Knowledge of differentially accumulated metabolites during AM symbiosis in cassava will facilitate the understanding of molecular interactions and provide metabolic markers for discriminating efficient symbiosis. Metabolomics analysis is an efficient and widely used molecular approach to assess the metabolome underlying organisms’ phenotype and investigate the variability of metabolites among different organs, varieties, and species of the same taxa (Scalbert et al., 2011; Dossou et al., 2021). Moreover, it helps understand biological processes and metabolic pathways (Farag et al., 2018; Dossou et al., 2022).

In the present study, we investigated the impact of AMF (*Rhizophagus irregularis*, DOAM197198) inoculation on cassava growth characteristics. We mainly examined metabolome and transcriptional changes during AM symbiosis in cassava *via* comparative metabolomics and transcriptomics analyses and unveiled key metabolites, pathways, and potential candidate genes. Our findings provide insights into AM symbiosis and valuable resources for cassava improvement.

## 2 Materials and methods

### 2.1 Materials and experimental layout

Huanan No. 9, an edible *Manihot esculenta* Crantz variety widely cultivated in China, and the fungus *Rhizophagus irregularis* (Ri), strain DOAM197198 were used as the host and AMF in this study.

Selected stems of Huanan No. 9, with the same thickness and size, were divided into uniform lengths (15cm). Next, the stem pieces were soaked in a mixed solution of acetamiprid, carbendazim, and acetamiprid to remove bacteria, fungi, eggs, etc. Then they were grown in pots (diameter of 15 cm) filled with a sterilized (high temperature of 121°C and high-pressure steam for 1 h) substrate at 2/3 height of the pot (**Figure S1**). The substrate was composed of a mixture of river sand and vermiculite at a ratio of 4:1. Prior to sown the pieces of stem in the substrate, about 800 spores (saturated in 1 g of distilled water) were spread on the surface of the substrate of the treatment group (to induce the AM symbiosis) and covered with an amount of the sterilized substrate until the stems could stand upright in the pot. An equal amount of sterilized substrate was added to the control group. In total, three groups, including two treatment groups (Ri, inoculated with the AMF) and one control group (CK, without inoculation), were formed. Each group was composed of 20 replicates. The Ri groups were Ri-3w (allowed to grow up to three weeks after inoculation) and Ri-6w (allowed to grow up to six weeks after inoculation). The pots were maintained in a greenhouse at 28°C and 16 h photoperiod (16 h light and 8 h darkness). All the pots (CK and Ri) were irrigated every week with 100 ml of low-phosphorus Hoagland nutrient solution (phosphorus content of 20 µM).

CK and Ri-3w root samples were collected three weeks after treatment, while Ri-6w roots were sampled after six weeks. The entire root system was cut off with scissors in clean Petri dishes and gently rinsed with tap water to remove the substrate. For each treatment, two groups of samples in three replications were prepared, one for microscopic observations and the other was immediately frozen-dried in liquid nitrogen, followed by storing at -80°C for metabolomics analysis and transcriptome sequencing. From our microscopic observations, the root starts to contact with the soil mycorrhizal layer and symbiosis occurs at 3 weeks, while a stable symbiosis occurs at 6 weeks. Therefore, the comparison between CK and Ri-3w is expected to show the changes of root response at the initial stage of symbiosis. The changes in root response from the initial stage of symbiosis to the stable symbiosis stage will be elucidated by comparing Ri-3w and Ri-6w samples. Samples of 3- weeks after inoculation were selected for metabolomics and transcriptomics analyses based on microscopic observations and to investigate molecular mechanisms at the initial stage of fungal hyphae colonization of cassava roots. The Ri-6w samples were taken to examine dynamic changes in the accumulation of DAMs and expression patterns of DEGs in inoculated plants.

### 2.2 Physiological parameter and mycorrhizal staining

At the sampling time, some physiological performances were evaluated, including plant height, stem thickness, root length, root weight (biomass), and leaf chlorophyll content. For the root biomass, root samples were oven dried at 60 °C for 72 hours. The total leaf chlorophyll content was measured on three fully opened leaves, with a SPAD (single-photon avalanche diode) meter on the plants. Three technical measures per leaf were conducted.

The WGA (wheat germ agglutinin) fluorescent dye staining method was used for the mycorrhizal staining directly after the samples’ collection. The root samples were introduced in a clean 50 ml centrifuge tube containing 10% KOH solution. Then they were fixed in FAA (formaldehyde alcohol acetic acid) solution for 24 h, followed by washing with distilled water. Next, root samples were placed in a water bath at 90°C for 5 minutes and then soaked again in a 10% KOH solution. After removing the KOH by rinsing three to five times with purified water, root samples were subsequently immersed in a 2% HCl solution, gently stretched using tweezers, left at room temperature for 15-30 minutes, and then washed four to five times in distilled water. Following, root samples were soaked in PBS buffer for 30 minutes; gently stretched with tweezers; and then washed to discard the PBS buffer. Finally, the root samples were placed on a clean 2 ml centrifuge tube, and a fresh mixture solution of PBS buffer and WGA488 (V:V = 1000:1) was added. The tubes were wrapped in tin foil paper and kept overnight in the refrigerator at 4°C. The following day, the symbiotic cells were observed using scanning electron microscopy (SEM, S-3000N, Hitachi Co., Ltd., Matsuda, Japan).

### 2.3 Metabolome profiling analysis

The widely targeted metabolomics profiling of root samples was carried out at MetWare Co., Ltd. (Wuhan, China). Briefly, 100 mg of each root sample (beforehand vacuum freeze-dried) was ground into a fine powder and dissolved in 1 mL of methanol (70%), vortex-mixed and extracted overnight at 4°C. Thereafter, extracts were centrifugated at 12,000 g for 10 minutes, and supernatants were collected and filtered with a 0.22 μm microporous membrane. All samples’ final extract was stored in a vial for subsequent metabolomics analysis. All sample extracts were mixed equally to form the quality control (QC) samples. The data acquisition system was a UPLC-MS/MS (ultra-performance liquid chromatography-mass spectrometry) platform composed of a UPLC (SHIMADZU Nexera X2, www.shimadzu.com.cn/) and a tandem mass spectrometry (MS/MS) (Applied Biosystems 4500 QTRAP, http://sciex.com/). The qualitative identification and quantification of metabolites; and multivariate analyses, including orthogonal partial least squares discriminant analysis (OPLS-DA), principal component analysis (PCA), hierarchical clustering analysis (HCA), differentially accumulated metabolites (DAMs) analysis, and functional annotation and enrichment analyses were conducted as per reported methods (Chen et al., 2013; Dossou et al., 2021; Dossou et al., 2022). Significant DAMs were filtered out at the thresholds of VIP (variable importance in projection) ≥ 1 and t-test *p* < 0.05.

### 2.4 RNA library preparation and sequencing

The total RNA extraction from cassava root samples, subsequent integrity and quality checking, sequencing on Illumina Hiseq platform, construction of cDNA library, and *de novo* assembly were carried out as per recently described methods (Sakamoto et al., 2019; Wang et al., 2020). Then the clean reads were mapped into the cassava reference genome (version 6.1, https://phytozome-next.jgi.doe.gov/info/Mesculenta_v6_1) (Bredeson et al., 2016), with the TopHat2 software (Kim et al., 2013). The parameter of no more than one mismatch was accepted in the alignment (Kim et al., 2013).

The expression level of each gene was normalized to the number of FPKM (Fragments Per Kilobase of transcript per Million reads) using the Cufflinks 2.0 software (Trapnell et al., 2012). The DEGs were identified using the DESeq2 software at thresholds of |log2Fold Change| ≥ 1 and *p*-value < 0.5 (Love et al., 2014). The functional annotations of DEGs were conducted *via* GO (gene ontology) and KEGG (Kyoto encyclopedia for genes and genomes) pathway enrichment analyses. The Blast2GO and KOBAS2.0 programs were used for the GO and KEGG analyses, respectively (Kanehisa and Goto, 2000; Conesa et al., 2005).

### 2.5 SYM Pathway

The establishment of mutualistic interactions between plant roots and beneficial microorganisms is governed by a common pathway (the SYM signaling pathway) which has been well characterized in *Medicago truncatula* (Bonfante and Genre, 2010; Genre and Russo, 2016). To identify SYM signaling pathway-related genes in cassava, *M. truncatula* SYM pathway genes were downloaded from the phytozome website and used for blast analysis against the cassava genome (Bredeson et al., 2016). Further, the DEGs related to this pathway were screened out as potential candidate genes for future studies.

### 2.6 Validation of the RNA-seq *via* quantitative real-time PCR (qRT-PCR)

To validate the transcriptome data, twelve genes with varying expression patterns were randomly selected for qRT-PCR analysis. The analysis was performed on LightCycler480 (Roche, Switzerland) real-time PCR system, with ChamQ™ SYBR1 qPCR Master Mix (Vazyme Biotech, Nanjing, China) (Song et al., 2021). The EF-1α (elongation factor 1-α) gene was used as the internal control. The specific primers of each gene are listed in **Table S1**. The expression level of each gene was computed *via* the 2¯^ΔΔCT^ method (Livak and Schmittgen, 2001).

### 2.7 Statistical analysis

Statistical analyses and graphing were conducted using GraphPad Prism v9.0.0121 (GraphPad 159 Software Inc., La Jolla, CA, USA). The data are presented as the mean ± SD, and statistical differences were achieved by t-test at *P* < 0.05. Heatmaps of gene expression patterns were carried out in TBtools software (Chen et al., 2020).

## 3 Results

### 3.1 Microscopic observations and impacts on physiological traits

**Figure 1:**
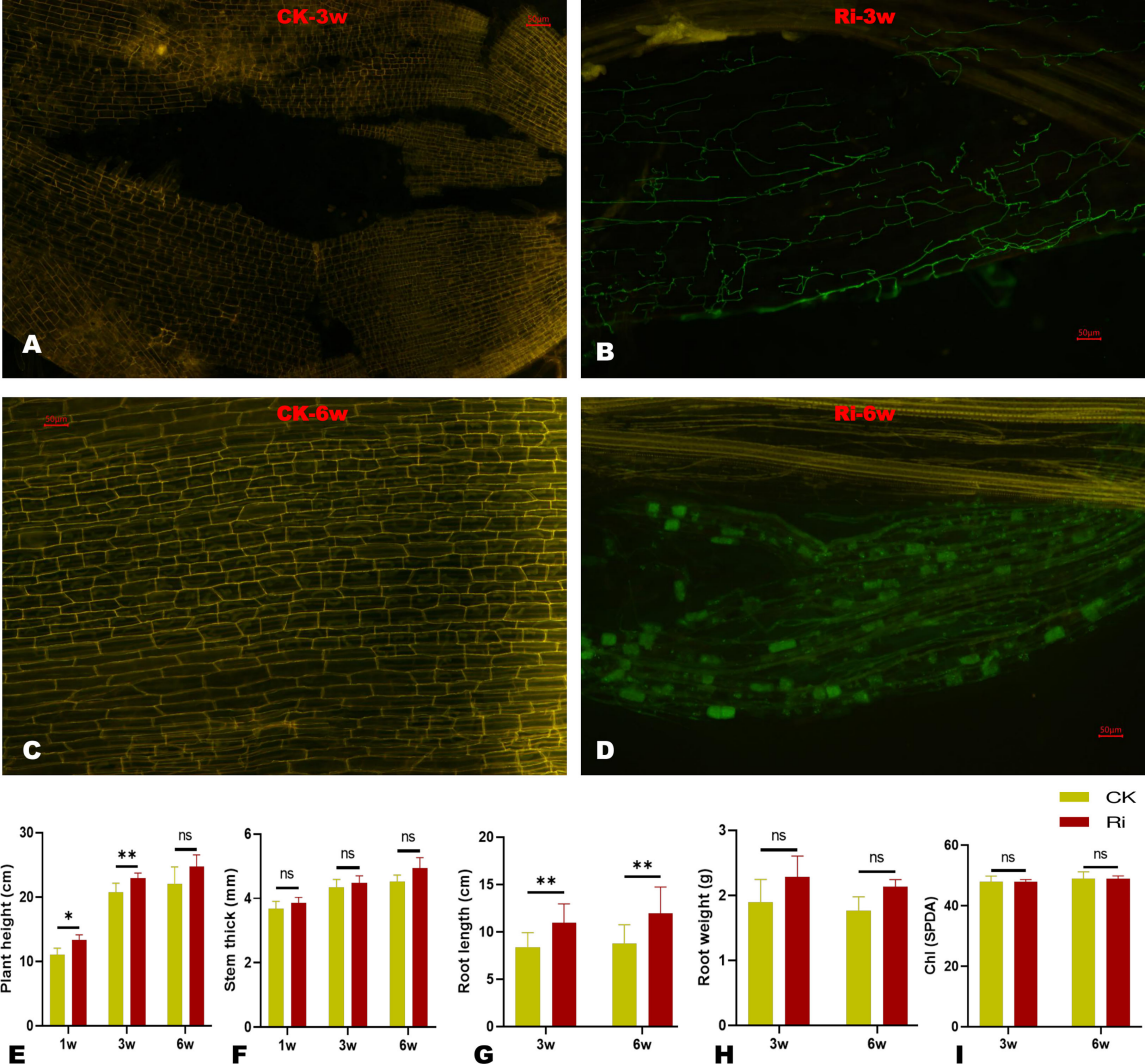
Microscopic observation and impacts of AM symbiosis on some physiological traits after three (3w) and six (6w) weeks of AMF inoculation. **(A, B)** Microscopic image of symbiotic cells at 3w of control (CK) and inoculated plants (Ri), respectively. **(C, D)** Microscopic images of symbiotic cells at 6w of control (CK) and inoculated plants (Ri), respectively. The green organelles in D and F indicate proliferating fungal hyphae. Pictures were taken at 10x, and scale bars indicate 50 µm. **(E–J)** Evaluation of some physiological traits, including plant height, stem thickness, root length, root dry weight, and leaf chlorophyll content, respectively. The data are presented as mean ± SD of three replicates. Comparisons were evaluated between CK and Ri at each time point using t-test at *P* < 0.05. * and ** indicate significant differences at *P* < 0.05 and *P* < 0.01, respectively. “ns” indicate not significantly different.

To confirm the establishment of AM symbiosis between the inoculated cassava roots and the fungi, we conducted microscopic observations on root samples at three and six weeks (3w and 6w) after inoculation. The results showed that symbiotic cells were already established at 3w, and proliferating fungal hyphae could be observed (**Figures 1A–D**). After 6w of inoculation, the fungal colonized a large surface of root cells, and hyphopodium could be observed (**Figure 1D**). As shown in **Figures S1A, S1B**, the AM symbiosis improved the physiological performances of inoculated cassava plants (Ri). Compared to the control plants (CK), the plant height and root length of Ri plants were significantly higher (**Figures 1E, G**). The stem thickness and root biomass (root dry weight) of Ri plants were slightly increased compared to CK (**Figures 1F, H**); however, the differences were not statistically significant. There was no difference in the chlorophyll contents of CK and Ri plants (**Figure 1I**).

### 3.2 Metabolites profiles of inoculated cassava plants

To examine the influence of AMF inoculation on cassava plants’ metabolism, we carried out metabolomics analysis of CK (3w) and Ri (3w and 6w) root samples. In total, 1016 metabolites, including 529 and 487 at the positive and negative ions, respectively, were identified and classified (**Table S2**). The main classes of metabolites identified were flavonoids (18.21%), followed by phenolic acids (16.83%), lipids (16.34%), amino acids and derivatives (10.43%), organic acids (8.07%), saccharides and alcohols (6.69%), alkaloids (6.50%), and nucleotides and derivatives (6.40%) (**Figure S2**).

**Figure 2:**
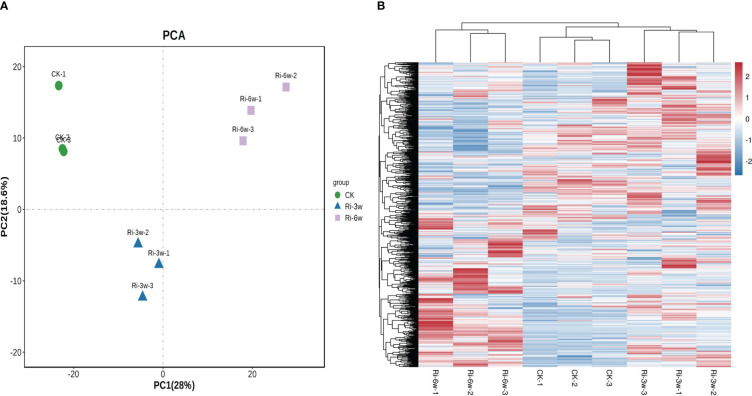
Principal component **(A)** and hierarchical clustering analyses **(B)** of asymbiotic (CK) and symbiotic (Ri) cassava roots based on their respective metabolite profiles. 3w and 6w indicate three and six weeks after AMF inoculation, respectively.

Hierarchical cluster analysis (HCA) and Principal component analysis (PCA) allow for exploring the variability of metabolites among samples of the same and different groups. As presented in **Figure 2**, the CK, Ri-3w, and Ri-6w samples were clustered separately on the PCA and HCA plots, indicating that their metabolite profiles were very different. Some metabolites were specifically induced in the roots of Ri plants (**Figure 2B**). To confirm the observed metabolite variation, we performed an OPLS-DA analysis. The results were supportive (**Figure S2**). We obtained strong goodness of fit (R2X > 0.643, R2Y> 0.997) and high predictability (Q2 > 0.777) of the models (**Figure S3**).

### 3.3 Differentially accumulated metabolites (DAMs)

**Figure 3:**
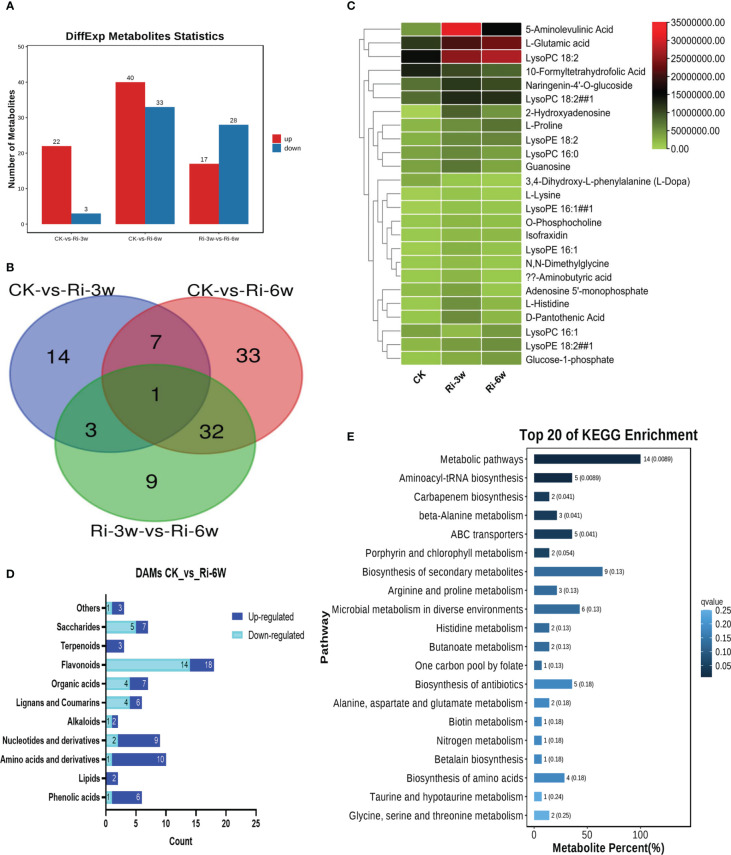
Differentially accumulated metabolites (DAMs) in pairwise comparison between groups and functional analysis. **(A)** Number of up- and down-regulated DAMs between groups. **(B)** Venn diagram showing the number of common DAMs shared by groups. **(C)** Heatmap of DAMs in between CK and Ri-3w. **(D)** Classification of DAMs between CK and Ri-6w. **(E)** KEGG annotation and enrichment result of DAMs between CK and Ri-3w.

In order to identify major metabolites involved in AM symbiosis in cassava, we performed DAMs analysis. Significant DAMs in pairwise comparison between groups were detected at thresholds of VIP ≥ 1 and *p*-value < 0.05. The volcano plots are shown in **Figure S4**. The results indicate that there were 25 (22 up-regulated) significantly differential metabolites in pairwise comparison between CK and Ri-3w (**Figure 3A**; **Table S3**). Meanwhile, 73 DAMs, including 40 up-regulated and 33 down-regulated, were identified between CK and Ri-6w (**Figure 3A**; **Table S4**). Venn diagram analysis showed that only eight DAMs overlapped between the two pairwise comparisons (**Figure 3B**), indicating a dynamic metabolome change in AM-inoculated cassava roots. Heatmap clustering analysis revealed that 5-aminolevulinic acid, L-glutamic acid, and lysoPC 18:2 were the most significantly induced metabolites in inoculated cassava (**Figure 3C**). The classification of the DAMs between CK and Ri-6w is shown in **Figure 3D**. It was noteworthy that 14 (14/18) flavonoids were down-regulated, while 7 (7/9) and 9 (9/10) nucleotides and amino acids, respectively, were up-regulated at 6w (**Figure 3D**).

To explore the molecular mechanisms involved in AM symbiosis in cassava, we carried out functional annotation and enrichment analyses of DAMs. The results showed that the DAMs between CK and Ri-3w were primarily involved in secondary metabolites biosynthesis, microbial metabolism in diverse environments, ABC transporters, aminoacyl-tRNA biosynthesis, biosynthesis of antibiotics, and amino acids metabolism (**Figure 3E**; **Table S5**). In addition to these pathways, the DAMs between CK and Ri-6w were mainly assigned to carbon and sugar metabolisms (**Figure S5**).

### 3.4 Roots’ transcriptome profiling of inoculated cassava plants

To gain insight into the molecular mechanisms involved in AM symbiosis in cassava, we conducted transcriptome analyses of CK, Ri-3w, and Ri-6w root samples. The summary of the transcriptome sequencing is presented in **Table 1**. The raw data and clean data ranged from 5.78 to 7.17 Gb and 5.71 to 7.07 Gb, respectively (**Table 1**). The Q20 varied from 97.45 to 97.76%, while the Q30 ranged from 92.95 to 93.68%. The clean reads were further mapped into the cassava reference genome, and the unique and total mapped reads varied from 74.18 to 90.99% and 75.71 to 92.86%, respectively (**Table 1**). These results show the high quality of the RNA-seq data. To examine the influence of AMF inoculation on cassava transcriptome, we conducted correlation and principal component analysis (PCA) of samples (**Figure S6**). The results revealed that the transcriptomes of CK and Ri-3w were not too much different. Samples of Ri-6w clustered separately and could be discriminated by PC1 (72.2%), suggesting a dynamic transcriptome change in inoculated cassava along with root development (**Figure S6B**).

**Table 1:** Summary of throughput and quality Illumina-based transcriptome sequencing of cassava roots during AM symbiosis.

Sample	Raw Data (bp)	Clean Data (bp)	Q20(%)	Q30(%)	GC(%)	Unique Mapped (%)	Total Mapped (%)
CK-1	6137843100	6057558629	97.6	93.29	43.55	90.99	92.86
CK-2	5787057900	5713669506	97.61	93.31	43.7	89.74	91.66
CK-3	6896868300	6804850731	97.72	93.61	43.76	89.51	91.41
Ri-3w-1	6503594400	6417997487	97.67	93.47	43.72	83.68	85.43
Ri-3w-2	7172058300	7075926943	97.61	93.33	43.6	89.85	91.76
Ri-3w-3	6403541100	6319452087	97.75	93.64	43.56	86.99	88.87
Ri-6w-1	6529975500	6450210439	97.51	93.05	43.41	74.18	75.71
Ri-6w-2	5948814300	5877235282	97.76	93.68	44.24	79.95	81.58
Ri-6w-3	6081218100	6018052399	97.45	92.95	43.82	85.12	86.87

### 3.5 Differentially expressed genes (DEGs) and functional annotation

**Figure 4:**
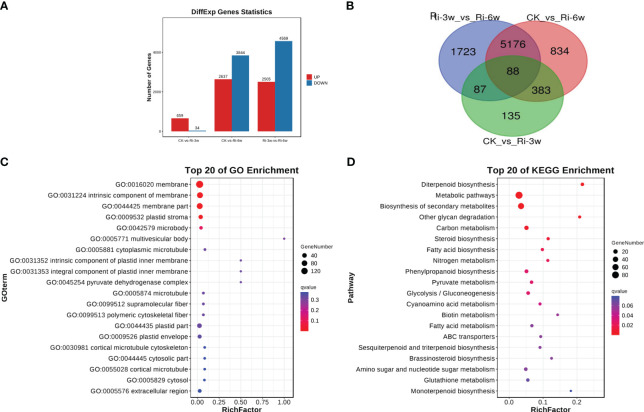
Differentially expressed genes (DEGs) and functional analyses. **(A)** Number of up- and down-regulated DEGs in pairwise comparison between groups. **(B)** Venn diagram showing the number of common DEGs shared by groups. **(C, D)** GO and KEGG annotation and enrichment analyses of DEGs between CK and Ri-3w, respectively.

To unveil key differentially regulated pathways, we first screened out DEGs in pairwise comparison between groups. The volcano plots of the DEGs in the comparison of CK against Ri-3w and Ri-6w are shown in **Figure S7**. We detected 693 DEGs, including 659 up-regulated and 34 down-regulated between CK and Ri-3w (**Figure 4A**; **Table S6**). Between CK and Ri-6w, there were 6,481 DEGs, of which 2,637 and 3,844 were up- and down-regulated, respectively (**Figure 4A**; **Table S7**). Of them, 88 DEGs were commonly identified in the pairwise comparison between all groups (**Figure 4B**).

We selected the DEGs in the pairwise comparison between CK and the other two groups for functional annotation and enrichment analyses. The most enriched GO terms that involve DEGs between CK and Ri-3w were related to membrane and plastid (**Figure 4C**). Meanwhile, DEGs between CK and Ri-6w were mainly associated with membranes, cell walls and junctions, and extracellular regions (**Figure S8A**). KEGG annotation and enrichment analysis of DEGs between CK and Ri-3w indicated that they are primarily involved in secondary metabolites biosynthesis, carbon metabolism, steroid and diterpenoid biosynthesis, phenylpropanoid biosynthesis, pyruvate metabolism, glycolysis/gluconeogenesis, fatty acid metabolism, and nitrogen metabolism (**Figure 4D**). The DEGs between CK and Ri-6w were mainly assigned to the biosynthesis of secondary metabolites, plant hormone signal transduction, phenylpropanoid biosynthesis, MAPK signaling pathway, plant-pathogen interaction, and metabolism of diverse sugars (**Figure S8B**). The lists of all identified pathways are shown in **Tables S8** and **S9**.

As we above reported, the 6w samples were taken in order to examine the dynamic changes in the expression levels of DEGs identified at 3w. Therefore, we mainly focused on DEGs between CK and Ri-3w for identifying key candidate genes underlying AM symbiosis in cassava.

### 3.6 Impact of AMF inoculation on phosphorus and nitrogen metabolisms

**Figure 5:**
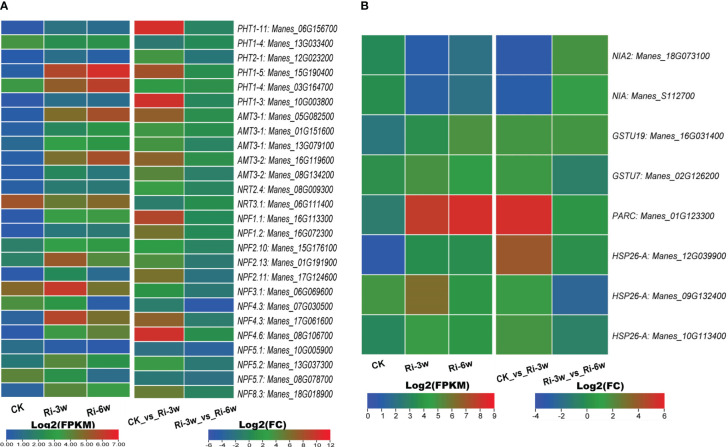
Transcription levels of DEGs related to nitrogen and phosphorus metabolisms. **(A)** Phosphorus and nitrogen transporters. **(B)** Other DEGs related to nitrogen metabolism. FPKM, Fragments Per Kilobase of transcript per Million mapped reads. PHT, inorganic phosphate transporter; AMT, ammonium transporter; NRT, high-affinity nitrate transporter; NPF, protein NRT1/PTR family-like. Genes’ annotation is presented in **Tables S9A, B**.

AM symbiosis promotes phosphorus and nitrogen uptake and metabolisms. We identified six inorganic phosphate transporters (PHT) and 20 nitrogen transporters related DEGs (**Figure 5A**; **Table S10A**). Of the PHT-related DEGs, *Manes_15G190400* and *Manes_03G164700* were the most significantly induced (**Figure 5A**). The identified nitrogen transporters included 5 and 15 ammonium (AMT) and nitrate (NRT) transporters, respectively. Both five AMTs were up-regulated, with *Manes_05G082500* and *Manes_16G119600* being the most highly induced (**Figure 5A**). Eleven of the NRTs were up-regulated, among which *Manes_16G113300*, *Manes_01G191900*, *Manes_17G061600*, *Manes_08G106700*, and *Manes_18G018900* were the most significantly induced (**Figure 5A**).

We also identified eight nitrogen metabolism related-DEGs, including two nitrate reductase (*Manes_18G073100* and *Manes_S112700*) and seven glutathione S-transferase family genes (**Figure 5B**; **Table S10B**). Both two nitrate reductase genes were down-regulated, while the glutathione S-transferase genes were up-regulated. Of them, *Manes_01G123300* (Log2(FC) = 5.5) and *Manes_12G039900* (Log2(FC) = 3.9) were the most significantly induced (**Figure 5B**; **Table S10B**).

### 3.7 Impact of AMF inoculation on sugars and phenylalanine metabolisms and other transporters

**Figure 6:**
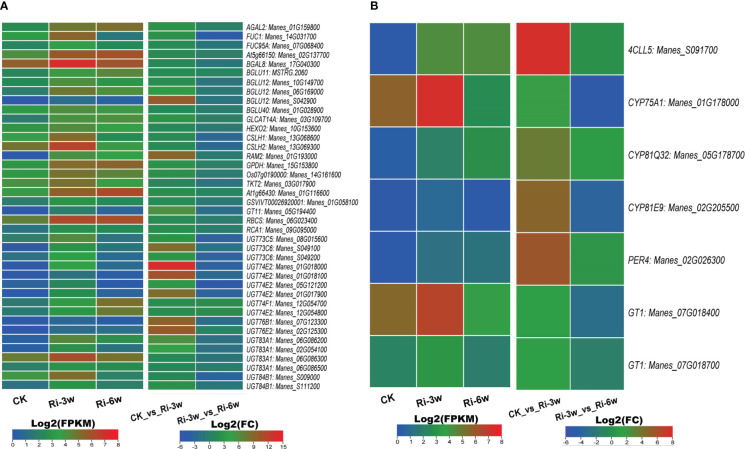
Transcription levels of DEGs related to sugar metabolism **(A)** and phenylpropanoid pathway **(B)**. FPKM, Fragments Per Kilobase of transcript per Million mapped reads. Genes’ annotation is presented in **Tables S9C, D**.

AM symbiosis influence sugar metabolism and phenylpropanoid biosynthesis. We identified 40 sugar metabolism and seven phenylpropanoid pathway-related DEGs (**Figure 6**; **Tables S10C, D**). It is noteworthily that all these DEGs were up-regulated in Ri at 3w (**Figures 6A, B**). Of the sugar metabolism-related DEGs, one beta-glucosidase (*Manes_S042900*) and six UDP-glycosyltransferases (*Manes_S049100, Manes_01G018000, Manes_01G018100, Manes_01G017900, Manes_07G123300*, and *Manes_02G125300*) were more than 8.5 folds significantly induced. The gene *Manes_S091700* encoding a 4-coumarate-CoA ligase-like was the most significantly induced (Log2(FC) = 7.72) in the phenylpropanoid pathway (**Figure 6B**).

Besides, 49 (four down-regulated) diverse other transporter family-related DEGs were also filtered out (**Table S10E**), and their expression patterns are shown in **Figure S9**. Of them, three ABC transporters (*Manes_11G015600*, *Manes_13G104000*, and *Manes_11G148900*), two acyl carrier proteins (Manes_03G055600 and Manes_16G087900), one oligopeptide transporter (*Manes_03G142900*), one ascorbate-specific transmembrane electron transporter (*Manes_17G086900*), one transmembrane protein (*Manes_15G046500*), four wall-associated receptors (*Manes_02G171700*, *Manes_11G070400*, *Manes_11G070300*, and *Manes_11G070200*), and two lipid transporters (*Manes_14G109400* and *Manes_S095100*) were more than 6.8 folds up-regulated in Ri at 3w.

### 3.8 Key transcription factors (TFs) and phytohormone-related DEGs

**Figure 7:**
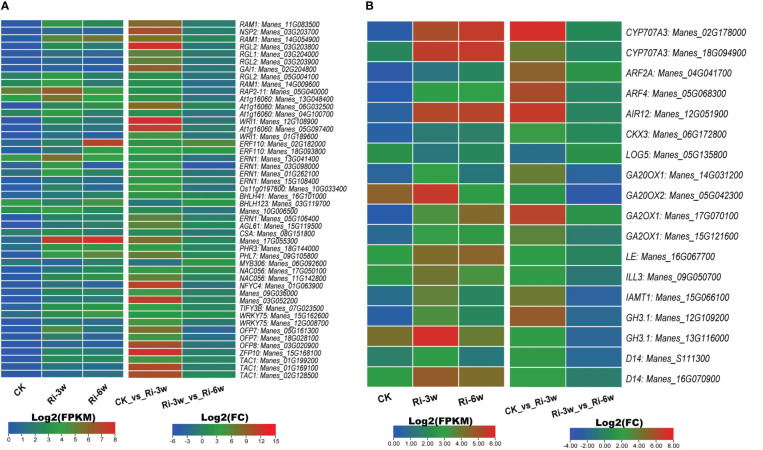
Transcription levels of DEGs belonging to transcription factor **(A)** and phytohormones **(B)** families. ABA, abscisic acid; AUX, auxin; GA, gibberellic acid; SL, strigolactones. FPKM, Fragments Per Kilobase of transcript per Million mapped reads. Genes’ annotation is presented in **Tables S9F, G**.

TFs and phytohormones play essential roles in AM symbiosis. Therefore, we screened out their respective DEGs and examined their expression profiles (**Figure 7**; **Table S10F**). The main differentially regulated TFs were DELLA (9 DEGs), EP2/ERF (13 DEGs), and MYBs (5 DEGs) (**Figure 7A**; **Table S10F**). As shown in **Figure 7A**, the genes *Manes_17G055300* (MYB), *Manes_05G040000* and *Manes_13G048400* (EP2), *Manes_13G041400* (ERF), *Manes_11G083500* and *Manes_14G054900* (DELLA), were the most induced TFs by the AMF inoculation.

We identified 18 phytohormone-related DEGs, including 2, 7, 2, 5, and 2 ABA (abscisic acid), auxins (AUXs), cytokinin (CYTs), gibberellic acid (GAs), and strigolactones (STs), respectively (**Figure 7B**; **Table S10G**). Most of them were highly induced, and only *Manes_05G135800* (CYT) was down-regulated in Ri at 3w (**Figure 7B**).

### 3.9 SYM signaling pathway-related candidate genes

To unveil key symbiosis (SYM) signaling pathway-related candidate genes in cassava, we performed a Blast search against *Medicago truncatula* SYM pathway genes. In total, we identified 41 genes homologous to *M. truncatula* SYM pathway genes, with a similarity ranging from 37.42 to 81.99% (**Table S11**). Thereafter, we examined their expression profiles and found that 28 of them were differentially regulated at least at one-time point. Accordingly, they were selected as potential candidate genes for further study on the SYM signaling pathway in cassava (**Table 2**). Among them, *MeSTR2, MeRAM1, MeKinG2, MeEXO70, MeABCB12, MeRAD1, MeHYP4, MeKinG1*, and *MeCYTB561* were more than 7 folds significantly induced in Ri at 3w.

**Table 2:** DEGs related to the SYM signaling pathway in cassava.

Genes	Log2(FC)		Annotation
	CK_vs_Ri-3w	CK_vs_Ri-6w	
*MeKinG2*	7.748	7.939	Cysteine-rich receptor-like protein kinase 3
*MeVAPYRIN*	NA	-2.157	Ankyrin-3-like
*MeABCB20*	NA	1.851	ABC transporter B family member 19
*MeEXO70*	7.063	6.586	Exocyst complex component EXO70A1-like
*MeEPP1*	NA	-2.730	Pyridoxal phosphate-dependent transferases
*MeRAD1*	NA	3.018	Scarecrow-like protein 21
*MeSTR2*	9.469	9.898	ABC transporter G family member 17-like
*MeRFCB*	6.573	7.192	Replication factor C subunit 3-like
*MeKinF*	6.691	6.579	Serine/threonine-protein kinase STY46-like
*MeGRAS*	2.361	NA	Protein SCARECROW-like
*MeKinG1*	3.326	2.529	Probable receptor-like protein kinase
*MeHYP3*	3.832	4.461	Protein prune-like protein
*MeFatM*	2.978	2.897	Palmitoyl-acyl carrier protein thioesterase
*MeSTR*	5.203	5.316	ABC transporter G family member 17-like
*MeABCB12*	7.649	6.756	ABC transporter B family member 19-like
*MeRAM1*	8.102	7.358	DELLA protein GAI-like
*Mepp2a*	2.652	NA	Perine/threonine protein phosphatase
*MeRFCA*	4.024	4.575	Replication factor C subunit 3
*MeRAM1*	NA	-3.404	Scarecrow-like protein 18
*MeTAU*	6.417	7.333	Clavaminate synthase-like protein
*MeFatM*	6.839	NA	Palmitoyl-acyl carrier protein thioesterase
*MeRAD1*	7.393	7.799	DELLA protein RGL1-like
*MeCCD*	NA	-2.901	Carotenoid 9,10(9’,10’)-cleavage dioxygenase 1-like
*MeHYP4*	7.668	NA	DUF538 domain-containing protein
*MeKinG1*	7.644	5.817	Probable serine/threonine-protein kinase PBL22
*MeCYTB561*	7.457	8.521	Probable ascorbate-specific transmembrane electron transporter 1

### 3.10 Validation of the transcriptome data through qRT-PCR

**Figure 8:**
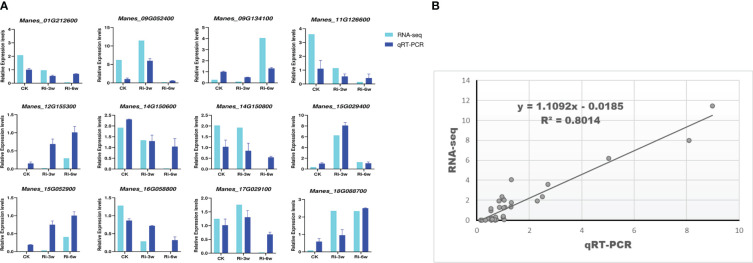
qRT-PCR validation of the RNA-seq data. Twelve DEGs were randomly selected for the qRT-PCR analysis. **(A)** Expression patterns of selected genes by RNA-seq and qRT-PCR. **(B)** Linear regression analysis of RNA-seq and qRT-PCR data. Relative expression indicates FPKM values for RNA-seq and the quantified expression levels by the 2¯^ΔΔCT^ method for qRT-PCR.

To confirm the reliability of the transcriptome data, twelve random DEGs were selected for quantitative real-time PCR analysis. The results showed that the expression patterns of these genes through the RNA-seq and qRT-PCR were consistent (R^2^ = 0.80; **Figure 8**), supporting the high confidence level of our findings.

## 4 Discussion

With regard to their huge beneficial effects on plant growth, productivity, adaptation, and resistance capability, there is an increasing interest in using AMF as bio-fertilizers to achieve sustainable agriculture and food security. A deep understanding of the molecular mechanisms and regulation of AM symbiosis is a prerequisite to attending this goal. Accordingly, this study combined metabolome and transcriptome analyses and explored the biochemical and molecular changes that occur during AM symbiosis in cassava.

The inoculation of cassava with AMF improved the plants’ physiological performances. For instance, plant height and root length were significantly increased in inoculated cassava plants after three weeks. These results are consistent with previous reports in cassava and other crops, indicating that AM improves plant biomass, growth, yield, and quality (Ceballos et al., 2013; Chen et al., 2017; Aliyu et al., 2018; Gao et al., 2020; Felföldi et al., 2022; Yan et al., 2022). The positive effects of AM on plants’ performances are due to the fact that AM improves water and mineral nutrient (primarily phosphorus and nitrogen) uptake and metabolism, which in turn stimulates primary and secondary metabolisms (Kaur and Suseela, 2020; Püschel et al., 2020; Fu et al., 2021; Saboor et al., 2021; Kaur et al., 2022; Rui et al., 2022). DAMs analysis revealed that AMF inoculation induced primary metabolism in cassava roots. The major DAMs were amino acids and derivatives, nucleotides and derivatives, lipids, and flavonoids. It is reported that lipid metabolism is stimulated during AM symbiosis as plants supply fungi with carbon, mainly in the form of photosynthate and lipids (Wewer et al., 2014). Moreover, it is found that fatty acid pathway is the key pathway that modulates the currency of exchange between AMF and cassava roots during symbiosis (Savary et al., 2020). Fourteen (14/18) flavonoids were downregulated in inoculated plants after six weeks, indicating that AM altered flavonoid biosynthesis in developing roots. This result may also suggest the catabolism of flavonoids *via* signaling or defense mechanisms. Indeed, flavonoids play important roles in mediating selective cross-talk between beneficial soil microbiomes and plants (Bag et al., 2022). The most DAMs were 5-aminolevulinic acid, L-glutamic acid, and lysoPC 18:2. Glutamic acid is essential for plant growth and development. It has emerged as a signaling molecule involved in various developmental processes, such as root architecture, seed germination, pollen germination, tolerance to abiotic stresses, resistance to pathogens, etc. (Qiu et al., 2020; Kim et al., 2021). 5-aminolevulinic acid modulates mineral nutrient uptake and enhances plant abiotic stress tolerance (Rhaman et al., 2021; Zhang et al., 2022). These findings infer that biofertilization with AMF may confer cassava plants abiotic and biotic stresses tolerance and improve production. Moreover, these key metabolites may represent key markers for discriminating efficient AM symbiosis in cassava.

Transcriptome analysis identified 693 and 6,481 DEGs in Ri at 3w and 6w, respectively. These DEGs and DAMs represent valuable resources for further study to better understand cassava biology, specifically its interactions with AMF. Functional enrichment analyses of DAMs and DEGs uncovered transport, amino acids and sugar metabolisms, biosynthesis of secondary metabolites, plant hormone signal transduction, phenylpropanoid biosynthesis, and plant-pathogen interactions as being the most significant differentially regulated pathways. Supportively, many transporter and phytohormone family genes and sugar metabolism-related genes were significantly up-regulated in inoculated plants. Previous studies have demonstrated that AM symbiosis significantly induces the expression of plant transporter genes (Porcel et al., 2016; Kameoka et al., 2019; Banasiak et al., 2021; Rui et al., 2022). Savary et al. have reported the significant up-regulation of the fatty acid biosynthesis pathway during AM symbiosis in cassava (Savary et al., 2020). Sugars and amino acids (primary metabolites) are precursors of diverse secondary metabolites and sources of energy. The identified phytohormone-related DEGs belong to ABA, AUXs, CYTs, GAs, and STs. These phytohormones are reported to play essential roles during AM symbiosis (Garrido et al., 2010; Gutjahr, 2014; Lanfranco et al., 2018; Faizan et al., 2020; Ho-Plágaro and García-Garrido, 2022a). STs are key signaling molecules used by plants to attract AMF through interactions with other phytohormones (Lanfranco et al., 2018; Faizan et al., 2020). GAs specific roles in AMF colonization and AM symbiosis are still unclear and confusing (Takeda et al., 2015; Tominaga et al., 2020; Tominaga et al., 2021; Tominaga et al., 2022). According to these studies, GA may promote or alter AMF penetration and proliferation in plants’ roots depending on the type of fungi. Two gibberellin-20-oxidase genes and three gibberellin 2-beta-dioxygenase genes were significantly up-regulated, suggesting they might modulate cassava plants and AMF symbiosis by regulating the expression of DELLA and other GRAS TFs. Gibberellin-2-oxidase was previously identified as a potential candidate gene for promoting AMF symbiosis in cassava (Savary et al., 2020). Therefore, functional characterization of these phytohormone-related genes is required to understand their regulatory effects on AM symbiosis in cassava.

It is worth noting that AM symbiosis improves phosphorus and nitrogen uptake and metabolism (Aliyu et al., 2018; Fu et al., 2021; Rui et al., 2022). We identified significantly up-regulated inorganic phosphate transporters (6), ammonium transporters (5), nitrate transporters (11), and glutathione S-transferases (6), and two significantly down-regulated nitrate reductase genes. The up-regulation of nitrogen and phosphorus transporters, coupled with the down-regulation of nitrate reductase in roots, indicate an improved uptake and a coordinated transport of these essential mineral nutrients from roots to the aboveground organs. The up-regulation of glutathione-related genes supports that AM symbiosis may improve cassava abiotic stress tolerance capability. Tremendous studies have demonstrated that AM enhances various biotic and abiotic stress tolerance abilities of plants (Sabra et al., 2018; Gao et al., 2020; Zhang et al., 2020; Jajoo, 2021; Lin et al., 2021; Jumrani et al., 2022).

The establishment of mutualistic interactions between plant roots and beneficial microorganisms is governed by a common pathway known as the SYM signaling pathway (Bonfante and Genre, 2010; Genre and Russo, 2016). This pathway has been well-studied in *M. truncatula* and rice (Harrison, 2005; Bonfante and Genre, 2010; Genre and Russo, 2016). It is mainly regulated by DELLA proteins, members of the GRAS TF family (Ho-Plágaro and García-Garrido, 2022a). Our analysis revealed that DELLA (9 DEGs), EP2/ERF (13 DEGs), and MYBs (5 DEGs) were the major differentially regulated TFs. These TFs might play critical roles in regulating AM symbiosis in cassava (Diédhiou and Diouf, 2018). The regulation of AM symbiosis in cassava involves complex mechanisms and is governed mainly by genetic variations in the genome of the two symbionts and genotypes × genotypes specifications (Mateus et al., 2019; Savary et al., 2020). Through blast analysis, we identified 28 significantly up-regulated SYM signaling pathway homologous DEGs in cassava. Twelve other significantly up-regulated SYM pathway ortholog genes have been identified in cassava (Mateus et al., 2019; Savary et al., 2020). These genes represent key resources to dissecting AM symbiosis regulatory network in cassava. Therefore, functional validation of all identified potential candidate genes is required to understand the regulation of AM symbiosis and uncover key markers and genes for genomics-assisted improvement of cassava. Particular attention should be given to the gene *MeRAM1* that encodes a DELLA protein GAI-like. *RAM1* was unveiled as the dominant gene in regulating the fatty acid pathway during AM symbiosis in cassava (Savary et al., 2020). Moreover, previous studies in other crops have shown that *RAM1* play central roles during AM symbiosis (Hohnjec et al., 2015; Rich et al., 2017; Hartmann et al., 2019; Ho-Plágaro and García-Garrido, 2022a).

## 5 Conclusion

Overall, this study provided a comprehensive data set by integrating metabolomics and transcriptomics analyses and enabled a global view of the complex biochemical and molecular changes that occur during AMF and cassava root symbiosis. DAMs and DEGs were identified, and Key differentially regulated pathways were revealed as being transport, amino acids and sugar metabolisms, biosynthesis of secondary metabolites, plant hormone signal transduction, phenylpropanoid biosynthesis, and plant-pathogen interactions. The AM symbiosis significantly stimulated nitrogen, phosphorus, and sugar metabolisms. In contrast, it altered flavonoid biosynthesis. GRAS (DELLA) TFs and some phytohormone family genes might be the key regulators of the two symbionts’ interactions. Potential candidate genes were uncovered for future functional studies. Our findings offer fundamental resources to dissect the regulatory network of the SYM signaling pathway in cassava and the efficient use of AMF in improving the crop production.

## Data availability statement

The datasets presented in this study can be found in online repositories. The names of the repository/repositories and accession number(s) can be found in the article/**Supplementary Material**.

## Author contributions

Conceptualization, YC and SC. Methodology, YG and ZP. Software, YG and SH. Validation, YG, YW. Formal analysis, YG. Investigation, YG, YW. and SZ. Resources, HL and JZ. Data curation, YG. Writing—original draft preparation, YG. Writing—review and editing, ZP. Visualization, WW. Supervision, YC and SC. Project administration, YC. Funding acquisition, YC. All authors contributed to the article and approved the submitted version.
