# Supplementary material for: Analysis of the molecular and biochemical mechanisms involved in the symbiotic relationship between Arbuscular mycorrhiza fungi and Manihot esculenta Crantz
Source: Front Plant Sci. 2023 Mar 7;14:1130924. doi: 10.3389/fpls.2023.1130924 (PMC10028151; doi:10.3389/fpls.2023.1130924)
Supplement: Supplementary file 1 [file DataSheet_1.pdf]

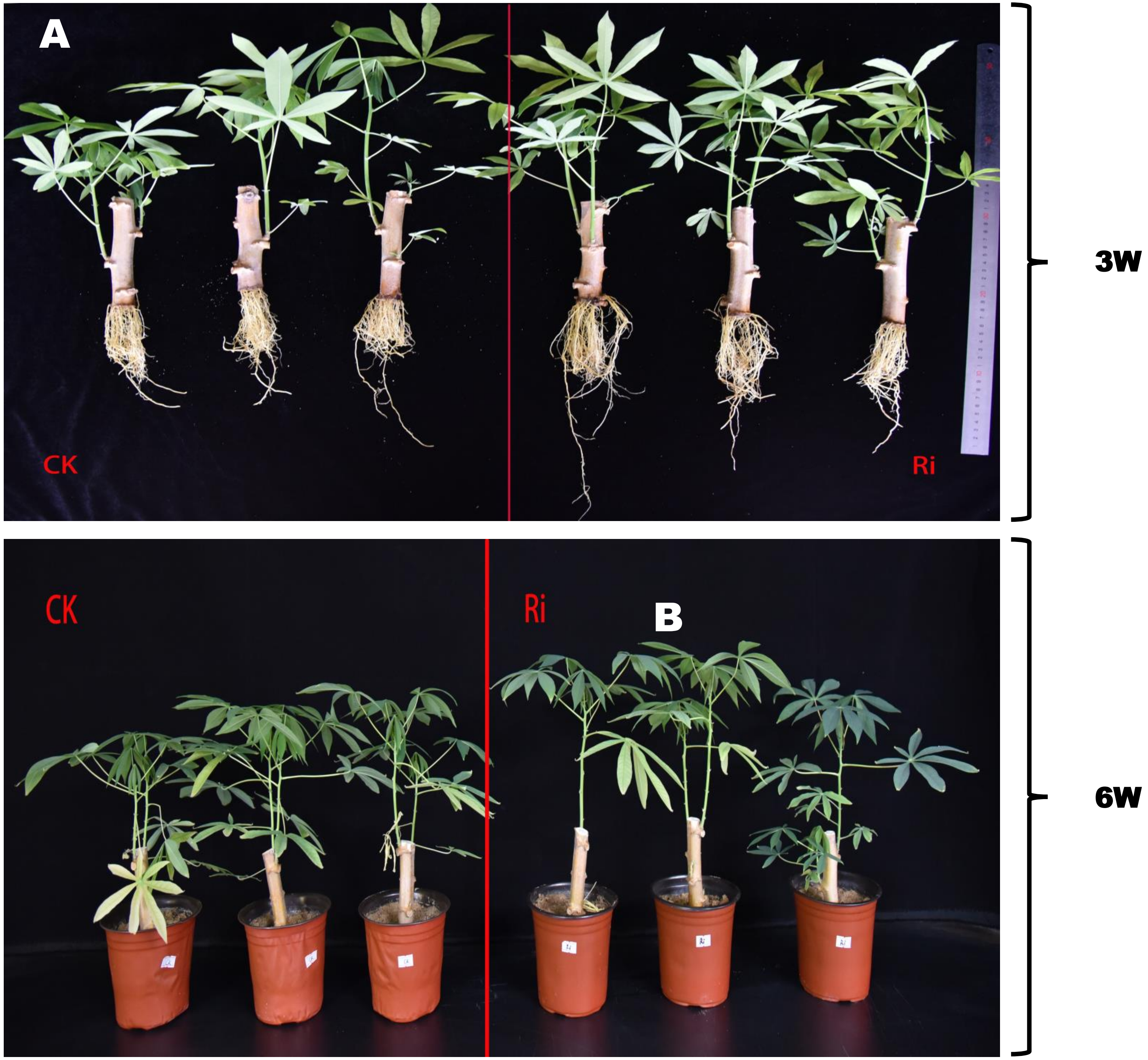

Figure S1. Morphology of plant at 3w and 6w, respectively.

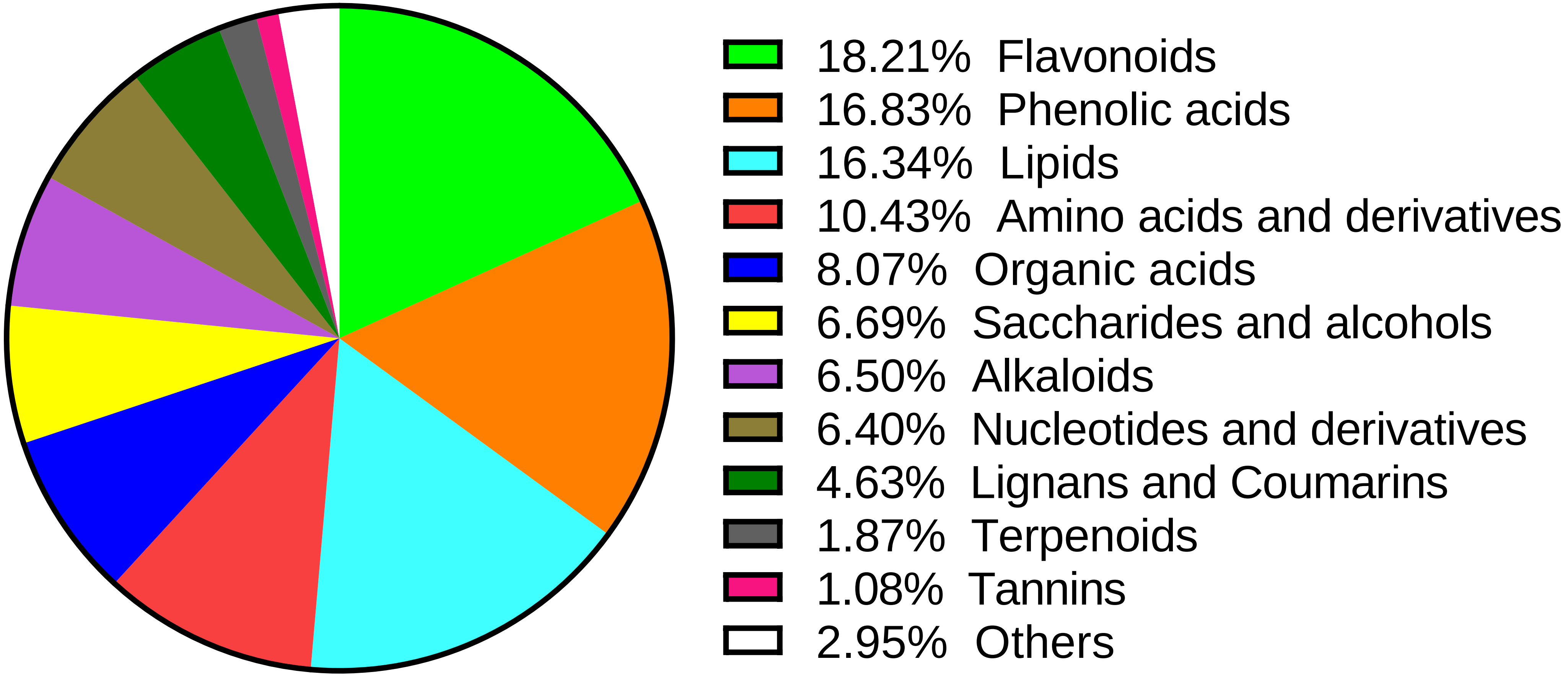

Figure S2. Classification of the 1,016 identified metabolites in cassava roots during AM symbiosis.

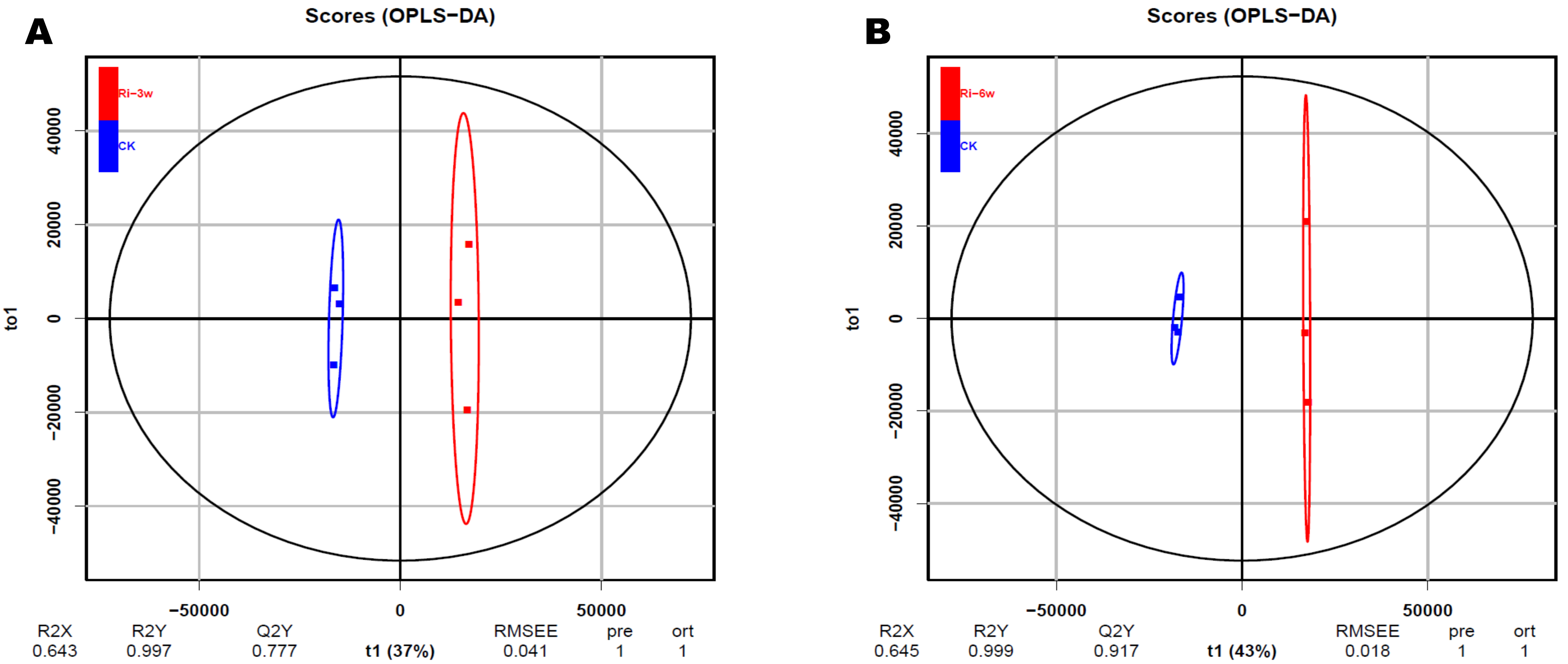

Figure S3. OPLS-DA analysis results of pairwise comparison of metabolite profiles of the control (CK) against AMF inoculated plants at 3w (A) and 6w (B), respectively.

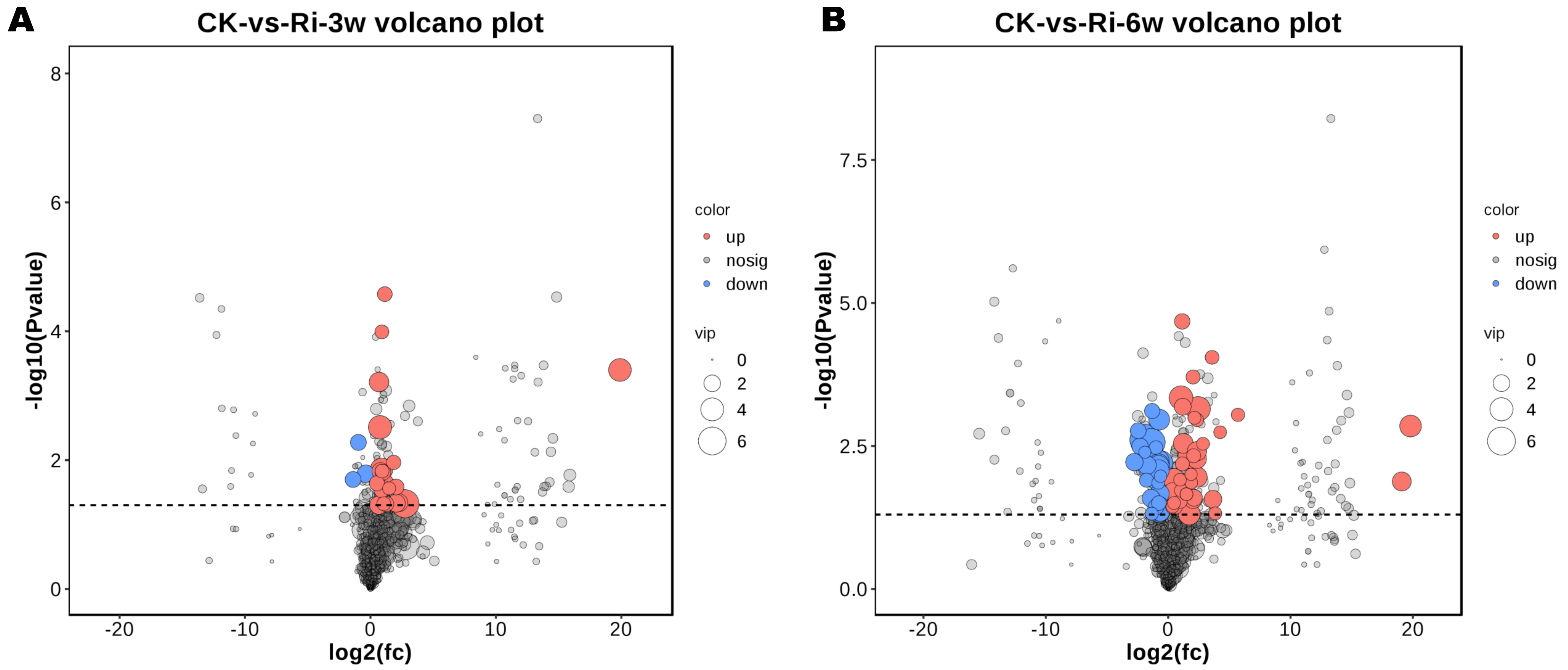

Figure S4. Volcano plots of DAMs in pairwise comparison of the control (CK) against AMF inoculated plants at 3w (A) and 6w (B), respectively.

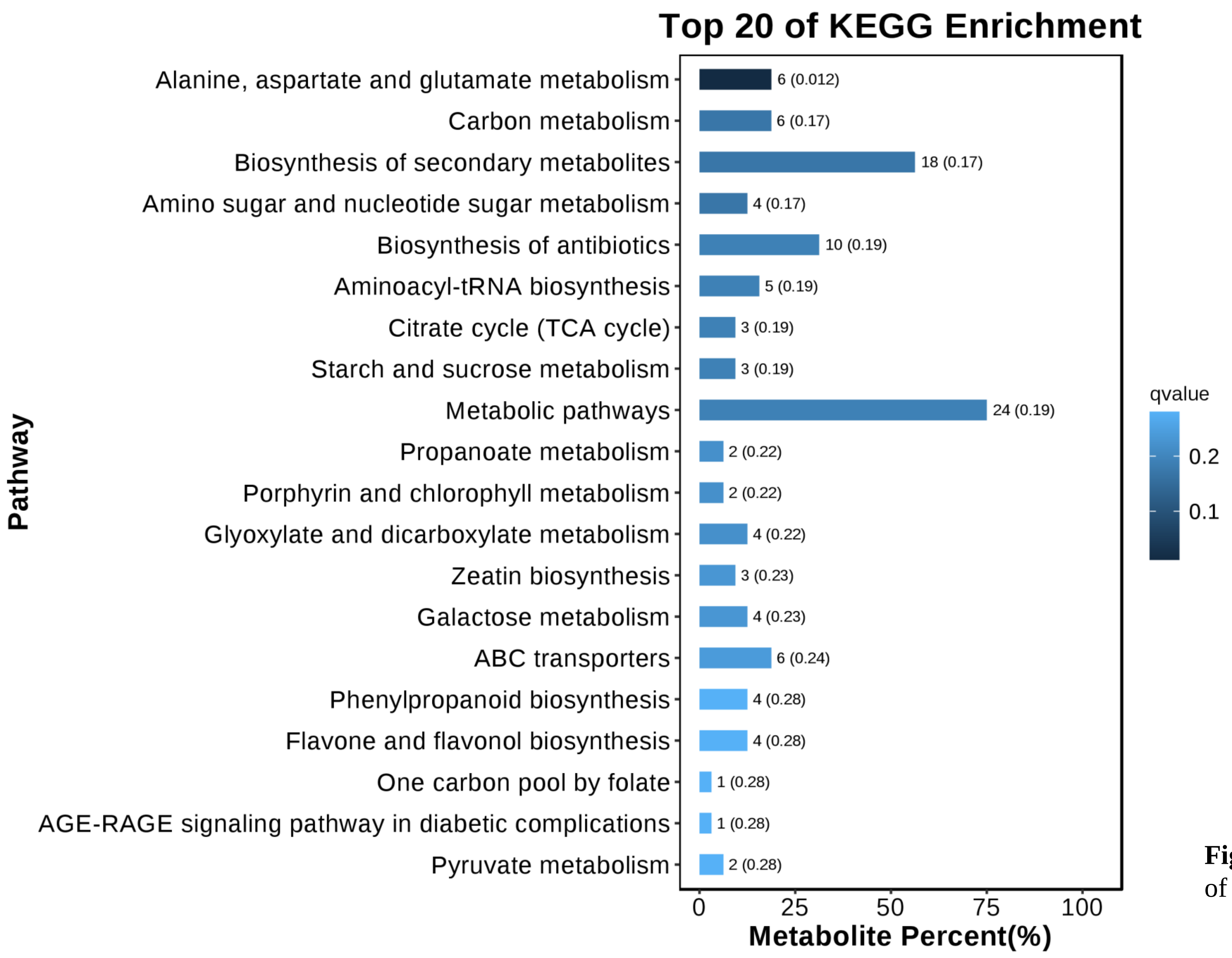

Figure S5. KEGG annotation and enrichment of DAMs between CK and Ri-6w.

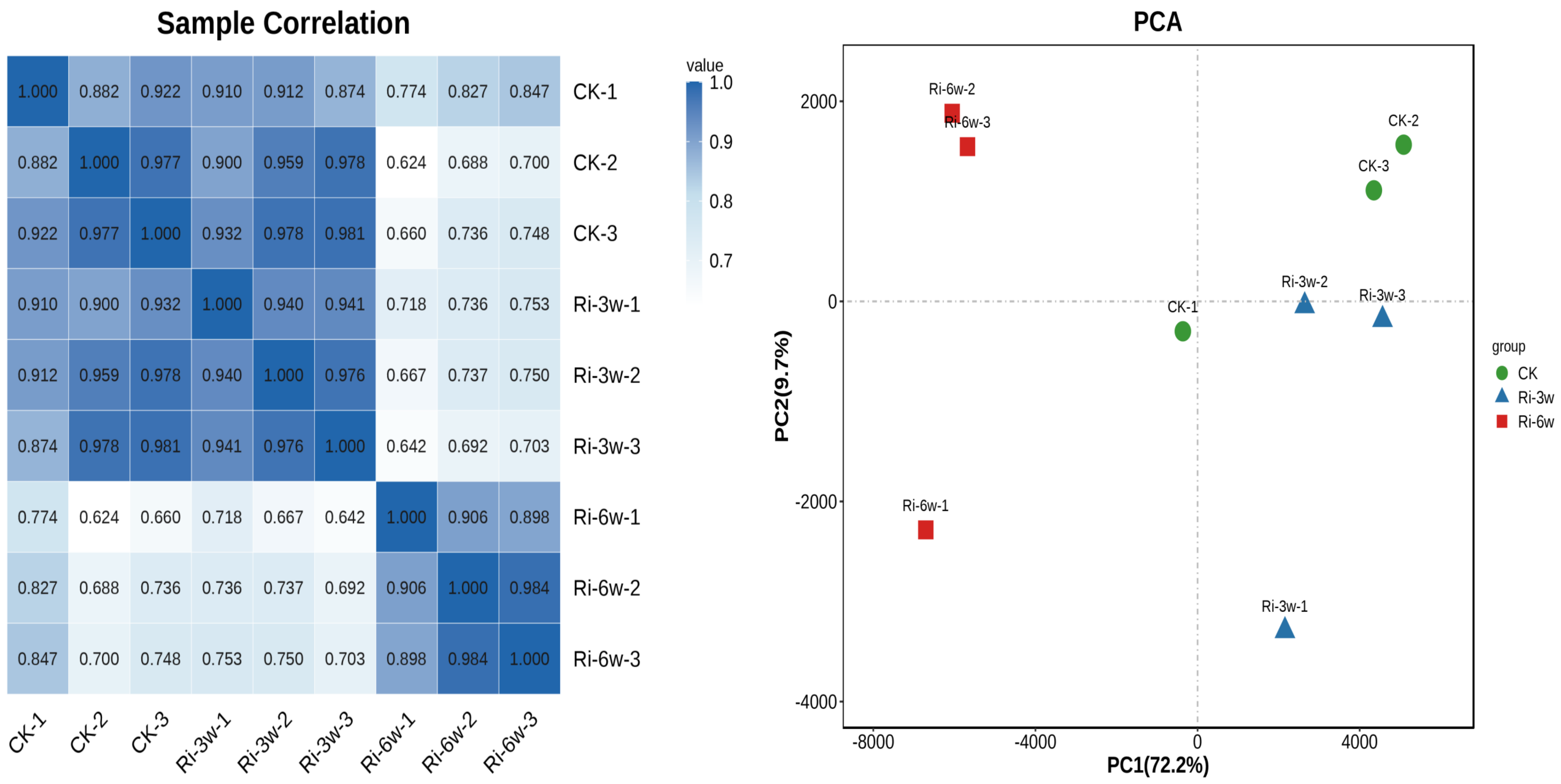

Figure S6. Correlation analysis (A) and principal component analysis (B) of cassava root samples during AM symbiosis based on the FPKM values.

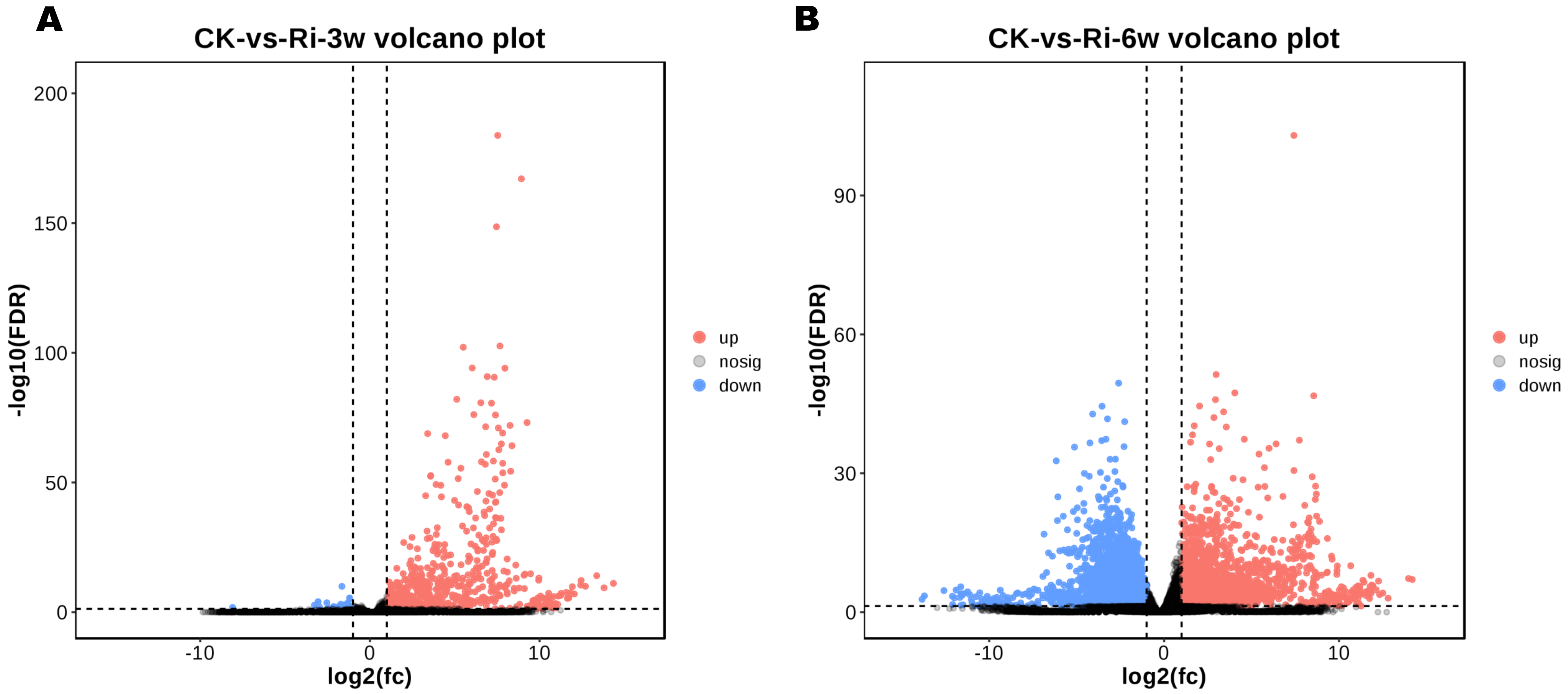

Figure S7. Volcano plots of differentially expressed genes (DEGs) in pairwise comparison of the control (CK) against AMF inoculated plants at 3w (A) and 6w (B), respectively.

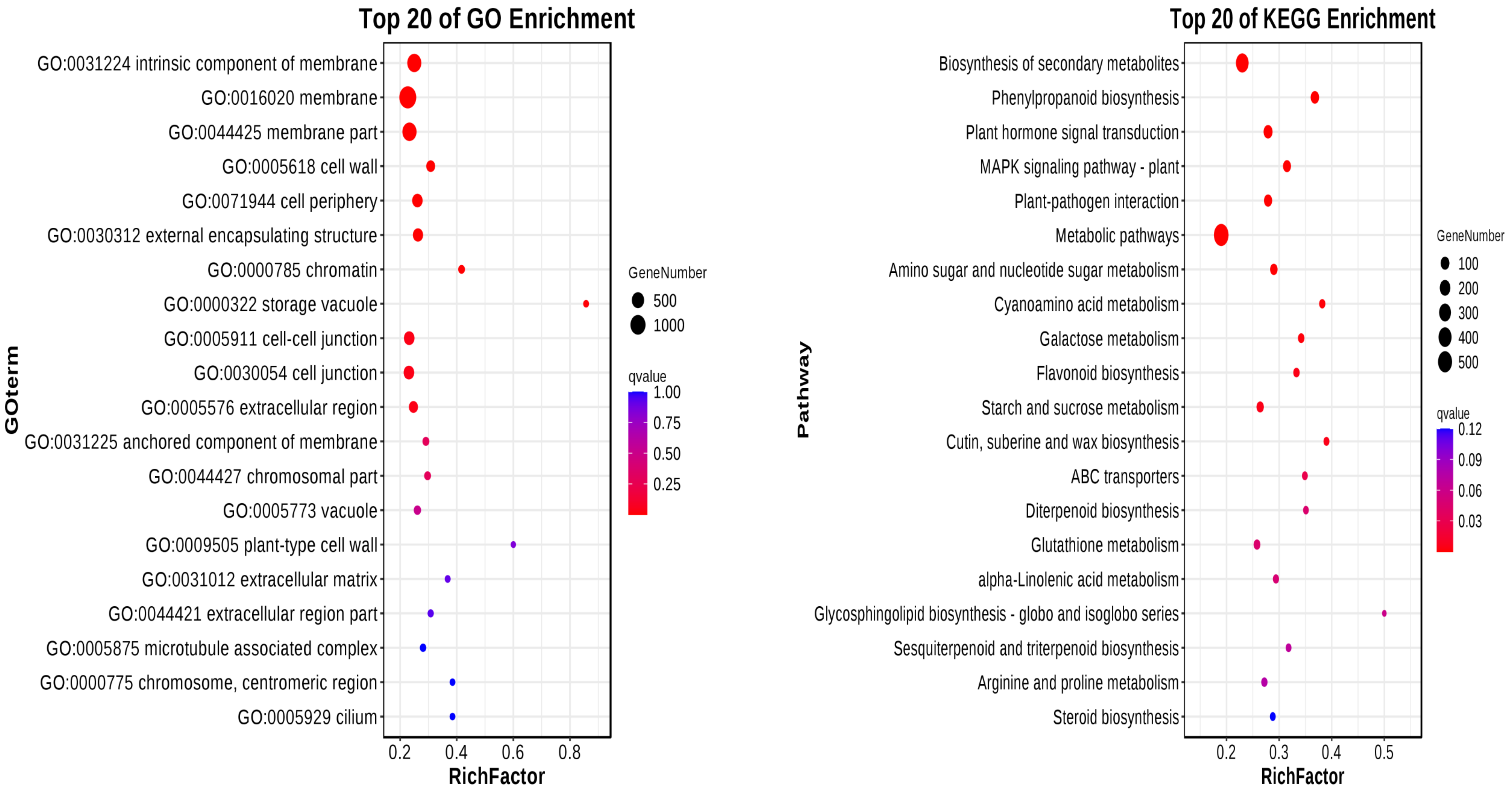

Figure S8. GO (A) and KEGG (B) annotation and enrichment analyses of DEGs between CK and Ri-6w.

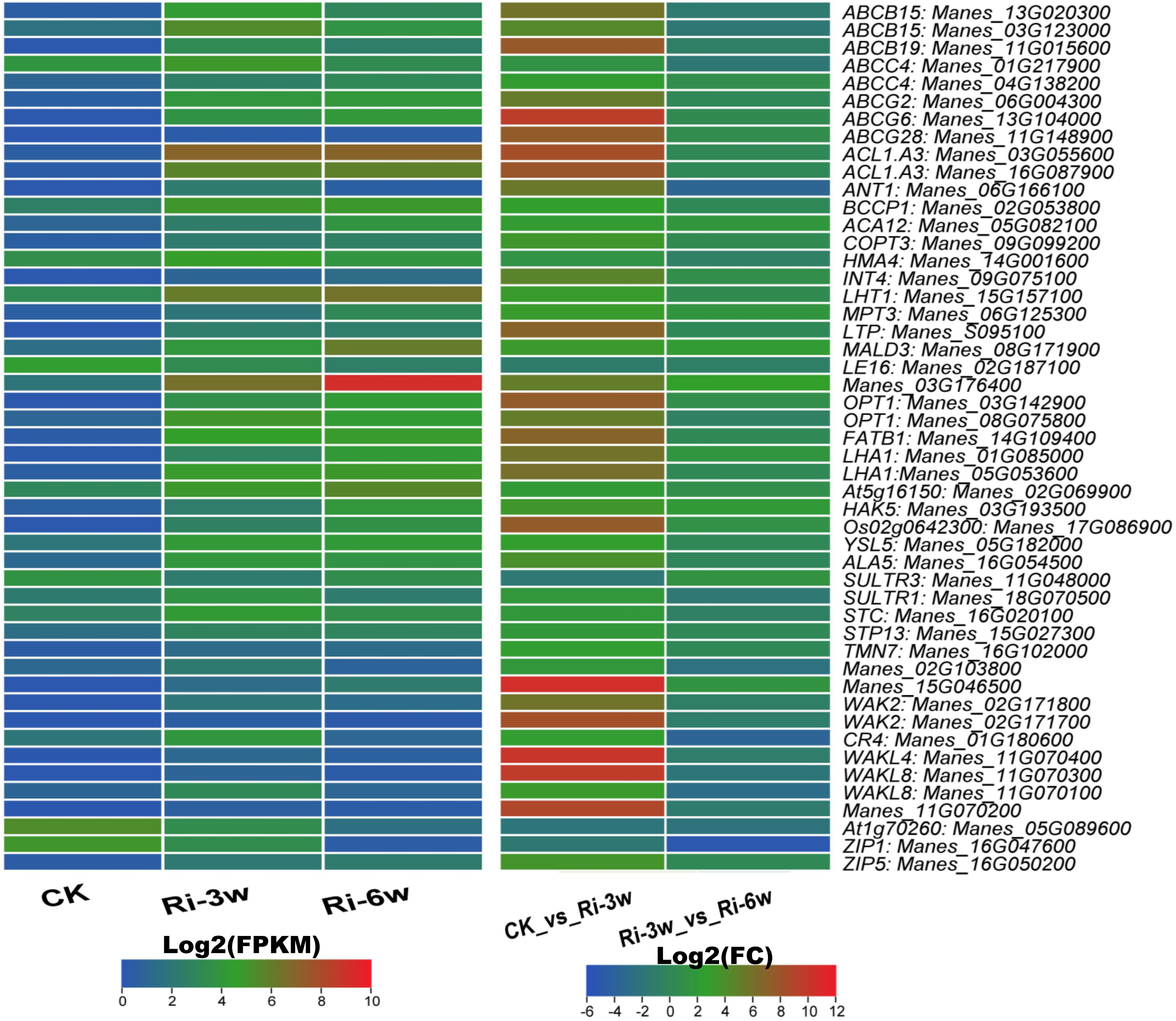

Figure S9. Transcription levels of diverse other transporter family-related the DEGs. Genes' annotation is presented in Table S9E.
